# Endoscopic Endonasal Transsphenoidal Treatment of Pituitary Apoplexy: Outcomes in a Series of 20 Patients

**DOI:** 10.7759/cureus.357

**Published:** 2015-10-20

**Authors:** Tong Yang, Fatema Bayad, Madeleine R Schaberg, Dimigtri Sigounas, Gurston Nyquist, Gregory Bonci, Kunal Patel, Apostolos John Tsiouris, Vijay K Anand, Theodore H Schwartz

**Affiliations:** 1 Neurosurgery, Weill Cornell Medical College, New York Presbyterian Hospital, New York; 2 Otolaryngology, Weill Cornell Medical College, New York Presbyterian Hospital, New York; 3 Radiology, Division of Neuroradiology, Weill Cornell Medical College, New York Presbyterian Hospital, New York; 4 Department of Otorhinolaryngology, Weill Cornell Medical College, New York Presbyterian Hospital, New York; 5 Weill Cornell Brain and Spine Center, Weill Cornell Medical College, New York Presbyterian Hospital, New York

**Keywords:** endoscopic endonasal transsphenoidal, hormone replacement, pituitary adenoma, pituitary apoplexy, visual defect

## Abstract

Objective: Pituitary apoplexy is a rare clinical entity and few cases treated with an endonasal endoscopic approach (EEA) have been reported. We report our experience of treating pituitary apoplexy using an EEA approach.

Methods: We performed a retrospective chart review on all the patients who underwent EEA skull base and pituitary surgery between December 2003 and March 2012 performed by the senior authors (THS and VKA) and identified patients with pituitary apoplexy. The extent of resection was determined volumetrically and the visual and endocrine outcome was evaluated.

Results: From a total of 488 skull base surgeries, there were 241 pituitary cases, of which 20 had apoplexy. The most common presenting symptoms included headaches (80%), endocrinopathy (95%), and visual symptoms (60%). Surgery was performed within 24 hours in 15% of patients, and > one month after ictus in 40% due to late referral. Gross-total resection (GTR) was achieved in 18 (90%) patients. There was one (5%) postoperative cerebrospinal fluid (CSF) leak treated with lumbar drainage. Of 12 patients with preoperative visual disturbances, seven had improvements. For those patients with visual field cuts, only 33.3% showed improvement. There was no postoperative visual deterioration. Two patients developed new transient postoperative diabetes insipidus (DI) but there was no new permanent DI. The mean duration of follow-up was 22 months (range: 6 days – 72 months).

Conclusion: The endoscopic endonasal transsphenoidal approach is an effective modality to treat pituitary apoplexy with a high rate of GTR and minimal risk. Delayed surgery may result in lower rates of visual field defect improvement.

## Introduction

Pituitary apoplexy is a rare but potentially life-threatening medical emergency. Typical presenting signs and symptoms often include sudden severe headaches, visual loss or decreased visual acuity, visual field cut, ophthalmoplegia, altered mental status, and impaired pituitary function [1-2]. It is generally considered a diagnosis based on symptoms and imaging; however, intraoperative and pathological findings of hemorrhage have been used to identify “subclinical (asymptomatic) apoplexy” [1, 3]. The severity of the symptoms can vary and the diagnosis is not always straightforward [1, 3-4]. The manifestations of pituitary apoplexy are attributed to the rapid expansion of an infarcted and/or hemorrhagic pituitary adenoma that has extended laterally into the cavernous sinus or superiorly to displace the optic chiasm and apparatus, with an occasional extension of the hemorrhage into the surrounding subarachnoid space [1-2]. MRI imaging is the most sensitive radiological modality for the detection/discrimination of acute and chronic intracranial hemorrhages and has been recommended for all suspected cases to provide information to guide management [5-7].

It is generally accepted that the indications for surgery following pituitary apoplexy are to prevent or reverse any significant visual deterioration, to potentially salvage damaged pituitary function, and to rescue patients with decreased consciousness, especially if symptoms are progressive. Although controversial, early surgery is advocated by some authors to achieve better postoperative results, depending on the specific preoperative symptoms [6, 8-10]. In the absence of significant or progressive visual symptoms, non-surgical management (i.e., hormone replacement) can be considered as the sole therapy. Although a few reports (with inherent patient selection biases) have shown equivalent post-treatment results between surgical and non-surgical management, particularly for the symptom of ophthalmoplegia, more patients are treated surgically in most reported series [4, 11].

A purely endoscopic endonasal approach has been established as an effective minimal access, natural orifice surgical procedure to treat pituitary tumors since the mid-1990s [12-15]. Several authors have advocated the endoscopic endonasal approach as a method to increase the extent of resection and decrease pituitary deficiency after surgery based on the increased visualization afforded by the panoramic and angled views provided by the endoscopes [12, 14-16]. However, reports of treating apoplexy with endoscopic techniques are limited [9, 17-18]. The aim of this study was to analyze the effectiveness of endonasal endoscopic transsphenoidal surgery at treating pituitary apoplexy in a series of consecutive patients treated by the senior authors (THS and VKA) and compare these results to historical reports in which a microscope was used. Special attention to visual outcome and timing of surgery was given as well as to the extent of resection and postoperative pituitary dysfunction.

## Materials and methods

This study was approved by the Institutional Review Board at Weill Cornell Medical College. Patient consent was waived.

A prospective database of all endoscopic endonasal surgeries performed by the senior authors (THS and VKA) between December 2003 and March 2012 was reviewed and any patients presenting with pituitary tumors, and specifically with apoplexy, were identified. Retrospective chart review was performed to collect clinical data, such as age, gender, presenting symptoms, pre- and postoperative pituitary function, visual function, pathology diagnosis, pre- and postoperative MRI findings, and durations of follow-up when available. Telephone calls were made to obtain up-to-date follow-ups with patients who did not have recent information in the chart.

All patients were evaluated with pre- and postoperatively MRI, except for two patients who only had preoperative CT scans (both had known pituitary tumors). The images were evaluated by a neuroradiologist (AJT) to determine the extent of resection using volumetric software using postoperative MRI scans obtained within 48 hours of the surgery. A GE Volume Viewer Plus version 5.013f workstation (General Electric, Princeton, NJ) was used for volumetric analysis. The software’s volume function was applied to the final composite construction.  The maximum tumor diameters reflect the single maximal diameter in any plane as measured on the GE workstations. Subsequent follow-up MRIs were obtained at three months and beyond. The images were evaluated by independent neuroradiologists without volumetric analysis.

For those patients who presented to the emergency room at the New York Presbyterian Hospital and were found to have adrenal insufficiency, high doses of hydrocortisone were given. Most patients had levels of pituitary hormones tested preoperatively. Postoperative pituitary hormone levels were typically checked on Day 2 after surgery and at subsequent follow-up, typically under the guidance of an endocrinologist, some of them were local referring physicians. If pituitary function did not improve at the time of the last follow-up (for those patients who were lost to follow-up), the dysfunction was considered permanent. Most of the patients with preoperative subjective visual complaints had pre- and postoperative neuro-ophthalmology examinations, although patients admitted through the emergency room were often not formally examined preoperatively by a neuro-ophthalmologist.

All patients underwent endoscopic endonasal transsphenoidal surgery with extended skull base approaches used as necessary [16, 19]. Surgical navigation was used in all cases and intrathecal injection of fluorescein (AK-FLUOR, Akorn, Lake Forest, Illinois) via lumbar puncture was used to identify potential intraoperative cerebrospinal fluid (CSF) leaks [20]. The absence/presence of an intraoperative CSF leak helped to determine the initial closure material used, i.e., Gelfoam (Pfizer, New York, New York) if there was no leak and autologous fat graft if a leak was present. The sellar defect was buttressed either with vomer or Medpor (Porex Corp., Newman, Georgia), and if tumors extended > 1 cm above the planum, a nasoseptal flap was used in cases operated on after 2008. Since 2005, we have been using Duraseal (Covidien, Hazelwood, MO) as a final layer followed by Floseal (Baxter, USA) [16]. Prior to 2005, we had used Tisseel (Baxter, USA) instead of the Duraseal.

## Results

### Patient demographics

From a total of 488 endonasal endoscopic skull base surgeries, there were 241 pituitary tumors; of these, 20 patients were identified who met the diagnostic criteria for pituitary apoplexy (i.e., sudden onset of symptoms and an intraoperative finding of hemorrhage within the adenoma). Patient demographics are presented in (Table 1).

**Table 1:** Clinical Characteristics F: Female, M: Male, values are reported as number (percent of the total 20 patients).

Demographics		
Gender	M	10 (50%)
	F	10 (50%)
Average age (range: years)	45.1 ( 12-71)	
Length of follow-up	6 days - 72 months (Mean: 22 months)	
Major preoperative signs & symptoms		
Headaches	16 (80%)	
Vision changes	12 (60%)	
Endocrinopathy	19 (95%)	
Potential precipitating factors		
Known pituitary lesions	7 (35%)	
Hypertension	9 (45%)	
Anticoagulation therapy/hypocoagulable State	4 (20%)	

### Clinical presentations

The most common presenting symptoms were headaches (80%), visual disturbances (60%) (including six with subjective decreased vision or blurry vision, six with visual field cuts, and three with diplopia), and pituitary dysfunction (95%; Table 1). Panhypopituitarism occurred in 45% and single axis endocrinopathy occurred in 35%. An elevated prolactin level was the most common hormonal abnormality, occurring in 45% (five with levels > 100 mg/L due to underlying prolactinomas and four with levels < 100 mg/L likely due to the stalk effect from the mass effect of the apoplectic event). There were two patients (10%) with diabetes insipidus (DI) (Tables 1-2). One patient presented with acute mental status change. All patients had identifiable ictus of sudden symptom onset. Seven patients (35%) had known pituitary lesions (two carried the diagnosis of prolactinoma and were being treated with dopamine agonists and two had previous resections at outside hospitals). Three patients were on anticoagulation therapy (clopidogrel, warfarin, or heparin drip) and one had a hypocoagulable state (thrombocytopenia due to chemotherapy for leukemia). Nine patients had known hypertension (Table 1).

**Table 2:** Visual Function and Endocrine Outcome N: No, Y: Yes, values are reported as number (percent of the total 20 patients) under Preop, values are reported as number (percent of the total patients with a particular abnormality/condition) under Postop. ^a^: Patient is lost to follow-up in spite of repeated effort to contact ^b^: Patient is lost to follow-up in spite of repeated effort to contact

Visual Function					
	Preop	Postop			
		Better	No change	Worse	Unknown
Decreased vision / Blurry vision	6 (30%)	4 (66.7%)	2 (33.3%)		
Visual field cut	6 (30%)	2 (33.3%)	4 (66.7%)		
Diplopia	3 (15%)	3 (100%)			
Endocrine Function					
	Preop	Postop			
		Better	No change	Worse	Unknown
Diabetes insipidus	N: 18 (90%)			2 (11.1%) transient	
	Y: 2 (10%)		2 (100%)		
Pan-hypopituitarism	N: 11 (55%) Y: 9 (45%)	3 (33.3%)	5 (55.6%)		1 (11.1%)^a^
Hypocortisolism	5 (25%)	3 (60%)	2 (40%)		
Hypothyroidism	2 (10 %)		2 (100%)		
Hyperprolactinemia	9 (45%)	8 (88.9%)			1 (11.1%)^b^

### Radiological imaging / evaluation

**Figure 1:**
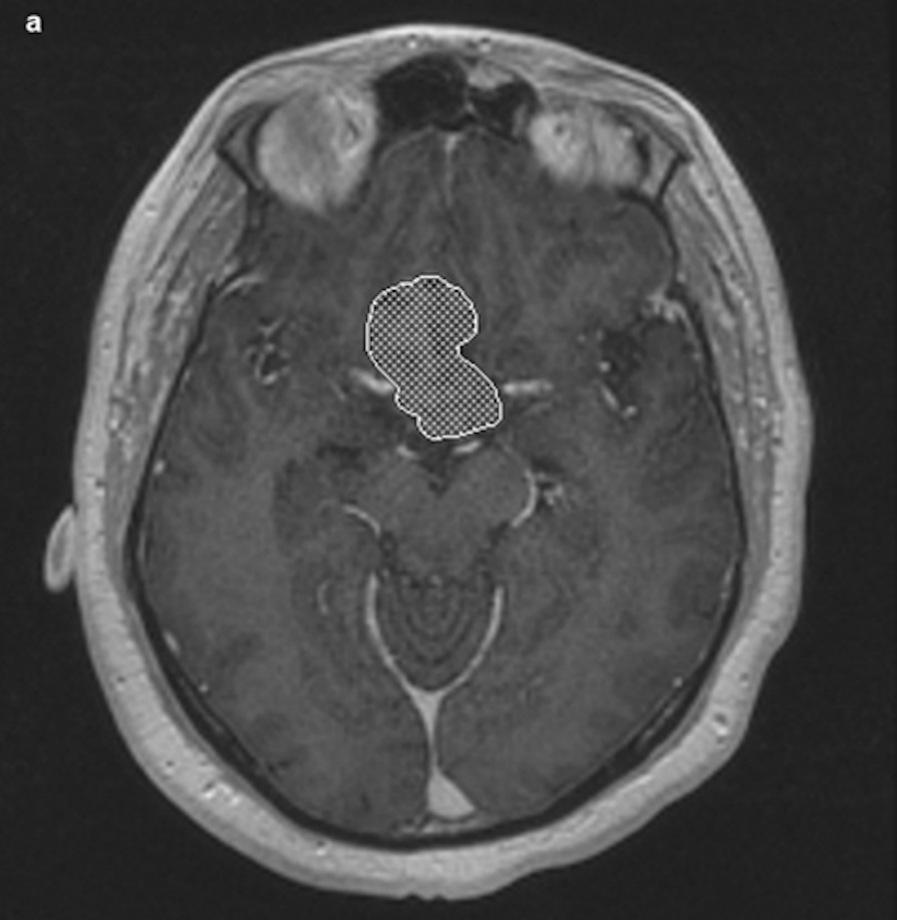
Representative axial spoiled gradient recall echo (SPGR) MR image of preoperative pituitary tumor post-volumetric analysis highlighting tumor silhouette

**Figure 2:**
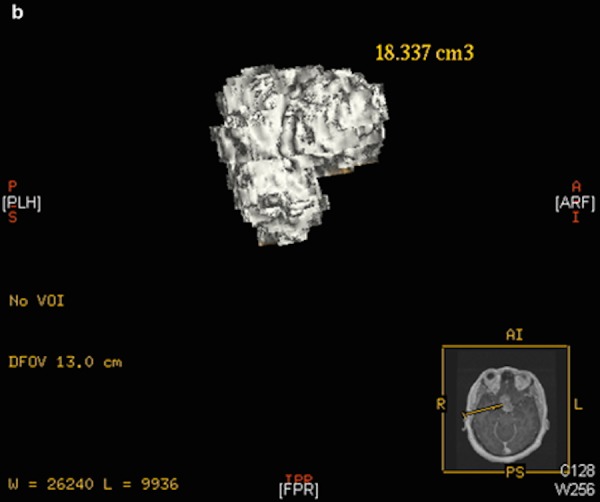
Rendered 3D adenoma reconstruction using software-calculated volumetric measurement

All 20 patients, except for two, had preoperative MRI. Seventeen of the 20 patients had both pre- and postoperative imaging performed at the New York Presbyterian Hospital/Weill Cornell Medical Center. Thirteen of these patients underwent a standardized volumetric pre- and postoperative MR imaging protocol to localize and assess tumor extent within two days of the surgical intervention (Figures 1-2).

The key sequence for the analyses in this study was an axially acquired 3D spoiled gradient recall echo (SPGR) T1-weighted (6.0/1.9 ms [TR/TE]), gadolinium-enhanced high-resolution MR image with 1.5 mm section thickness. MR imaging was obtained on either 1.5 Tesla (T) or 3.0 T MR imaging units using GE 15M4a acquisition software (Signa HDx). The intravenous contrast used was Magnevist (Bayer Pharmaceuticals, Wayne, NJ) dosed by weight at 0.2 mL/kg. Two patients underwent post-contrast MR brain imaging protocols without the 3D SPGR imaging.  In these cases, the imaging sequence of interest was an axially acquired T1-weighted (650/8.6 ms [TR/TE]), gadolinium-enhanced MR image with 5 mm section thickness. Finally, two patients had only preoperative CT scans available for analysis; these patients underwent a standard institutional contrast-enhanced head CT protocol on a 16 or 64 detector CT-scanner (Discovery CT750HD and Lightspeed Xtra; GE). Acquisition parameters were as follows: 140 KV, 190 mA, and section thickness of 1.25 mm with no gap. Images were acquired axially and reformatted in the sagittal and coronal planes at 1.25 mm. Omnipaque-300 at a standard dose of 75 mL intravenously was the contrast agent used in the CT scans. 

Across all imaging modalities, a direct comparison was made between matched pre- and post-contrast axial sequences. In the MR images, acute and subacute hemorrhage characteristically exhibited T1 hyperintensity on pre-contrast imaging with minimal or no enhancement on post-contrast imaging, consistent with previous descriptions of intratumoral hemorrhage [21-22]. The tumor was distinguished from normal pituitary by virtue of its morphology and hypo-enhancement on post-contrast T1-weighted images. For the two patients with preoperative CT imaging, hemorrhage was identified due to its relative hyperdensity compared to adenoma. In 16 cases, the hemorrhage obliterated or displaced the adenoma to such a degree that it was not possible to separate the components (tumor versus hemorrhage) for volumetric measurement.

The extent of chiasmal compression by the lesion on preoperative MRIs combined with the patient’s symptoms helped to determine the urgency of the surgery. Not all patients who had radiographic evidence of chiasmal compression showed visual symptoms. Postoperative MRIs were used to evaluate the extent of surgical resection as well as to monitor recurrence/progression during follow-up visits. Twelve patients have had follow-up MRIs at three months or longer after surgical resection (including one patient after repeated surgery as described below). The MRI scans were evaluated by independent neuroradiologists, but no volumetric studies were performed.

### Timing of surgical intervention

Three patients were operated on within 24 hours of presentation due to sudden significant visual disturbances refractory to steroid treatment. Four patients were treated within one week. Two were treated within two weeks of symptom onset, and three were treated within a month of the identifiable ictus of symptom onset. Eight patients had operations more than a month after the initial onset of symptoms, all of whom were either referred to our institution at this time point or had significant medical comorbidity to prevent more urgent intervention.  

Volumetric imaging of postoperative MRI indicated that gross total resection (GTR) was achieved in 18 patients (90%) and two patients had a subtotal resection (STR). One patient had persistent fluid signal within the sellar region on follow-up MRI with concurrent compression of the chiasm; a second endoscopic resection was performed eight months later to remove intralesional evolving hemorrhagic fluid (Table 3). The other patient had a 90% resection (preoperative tumor volume: 13.956 cm^3^, postoperative tumor volume: 1.405 cm^3^). No other recurrence or progression was noted within our follow-up time frame (mean: 22 months; range: 6 days -72 months). For the twelve patients who have had follow-up MRIs at or beyond three months after surgical resection, no radiographic evidence of recurrence was detected, and all have had GTR based on the volumetric studies of the postoperative scans obtained at 48 hours after surgery.

**Table 3:** Surgery-related Information Values are reported as number (percent of the total 20 patients).

Time to surgery from the point of symptom onset	
<=1 day	3 (15%)
>1 day but <=1 week	4 (20%)
>1 week but <=2 weeks	2 (10 %)
>2 weeks but <=1 month	3 (15%)
>1 month	8 (40%)
Extent of resection (base on pre- & postoperative MRI imaging)	
Gross-total resection	18 (90%)
Subtotal resection	2 (10%)
Surgery-related complications	
Cerebral spinal fluid leaks	1 (5%)
Postoperative meningitis / confusion (transient)	2 (10%)
Postoperative epistaxis	1 (5%)

### Pathological findings

The pathological findings are summarized in Table 4. No pathology was recorded for the patient who had persistent fluid signal and chiasmal compression following the first surgery. Pathological evaluations showed hemorrhagic fluid without viable tumor present at the second surgery. The majority of patients (80%) harbored histologically-proven pituitary adenomas: 40% were non-secreting and 25% were prolactinomas. No viable tumor, except for necrotic debris, was found in four patients, although they were presumed to have pituitary adenomas in which the hemorrhagic destruction of the tumor rendered the histology non-diagnostic.

**Table 4:** Pathological Findings Values are reported as number (percent of the total 20 patients).

Pituitary adenomas: 16 (80%)
Non-secreting: 8 (40%)
Prolactin: 5 (25%)
Adrenocorticotrophic Hormone: 1 (5%)
Follicle Stimulating Hormone/Luteinizing Hormone: 2 (10%)
No viable tumor seen, only necrotic debris: 4 (20%)

### Visual outcome

Twelve patients presented with visual symptoms. Ten complained of visual loss (subjective or proven with formal visual field exam) and three had diplopia. Four of the six patients with subjective decreased visual acuity did not have documented formal visual fields; some of them were taken to the operating room before formal visual field testing could be completed given the urgent or semi-urgent nature of the surgery. The three patients with diplopia improved. Of the six patients with formal preoperative visual fields, only two had documented improvement (one operated on within 24 hours and the other after one month of symptom onset). For the four patients without improvement of the visual fields, one was operated on within 24 hours and the other three after one month of symptom onset. Of the patients with subjective blurry vision, four had improvement. No visual deterioration was noted (Table 2).

### Endocrine outcome

The two patients who presented with DI did not improve after surgery (surgeries performed <1 week and >1 month after symptom onset). Two patients developed new postoperative DI, both of which were transient. Nine patients (45% of 20) presented with panhypopituitarism, three of which (33.3%) improved after surgery. Time to operation in these patients varied from one week to greater than a month. Five patients had persistent deficits (one was operated on within 24 hours, three within two weeks, and one after one month) and one patient’s status was unknown (lost to follow-up in spite of repeated effort to contact). Three out of the five patients that presented with isolated hypocortisolism showed improvements (operated on from less than a week to greater than a month from the onset of symptoms) and did not need hormone replacement; two had persistent deficits (time to surgery was less than one day or one month). Two patients with isolated preoperative hypothyroidism showed no improvement. Nine patients showed hyperprolactinemia preoperatively due to either the underlying prolactinomas (five with prolactin level > 100 mg/L) or stalk effect from the mass lesion. Eight (88.9%) had resolution and did not need dopamine analog treatment (except for one still on cabergoline). The long-term status of the other one was unknown due to lost to follow-up (Table 2).

### Complications

There was one postoperative CSF leak, which was managed with five days of lumbar drainage. There were two cases of postoperative confusion which resolved spontaneously. One patient had persistent intermittent epistaxis during the immediate postoperative period and was managed at the otolaryngology clinic (Table 3).

## Discussion

The sine qua non clinical manifestations of pituitary apoplexy are sudden headaches, visual disturbances, and endocrine dysfunction. This condition can be difficult to diagnose unequivocally due to variations in symptomatology and the degree of severity at presentation as well as possible mimicry of other disease processes. Radiological imaging studies, intraoperative findings, and pathological examinations, together with patients’ symptomatology, are usually combined to confirm the diagnosis retrospectively [1]. The general consensus is that pituitary apoplexy is a clinical diagnosis most likely resulting from the hemorrhage/infarction of an underlying pituitary tumor and subsequent sudden and fulminant volume expansion [1-2]. The reported incidence of pituitary apoplexy varies from 0.6% to 27.7% [2]. However, the exact rate of occurrence is difficult to obtain due to the lack of a precise definition of the disease. Controversy exists over whether asymptomatic apoplexy should be considered as true apoplexy [1, 3].

All of our patients had an identifiable ictus of symptom onset after review of their medical history; however, for some of them, the symptoms were not severe enough for the patients to seek immediate medical care. As a result, some of our patients were referred to us after lengthy workups at outside facilities, and the duration of time from the onset of the symptoms to arrival at our institution could be weeks to months. This is not unique to our practice. A range of “a few hours to 80 days” of time-to-presentation was noted in another larger series of apoplexy patients [4]. The optimal time for surgical intervention is still under debate, although many advocate for early intervention [6, 8-10]. Our decisions on when to operate have been based on the severity and rate of progression of a patient’s visual symptoms since hormonal deficiencies can be treated medically. Likewise, intractable headaches may require sellar decompression for relief. As a result, the duration of time from the onset of symptoms to surgery varied widely in our series. Sixteen patients (80%) had pathologically confirmed underlying pituitary adenomas, and 10 of them (62.5%) had the apoplectic event as the initial presentation without a prior diagnosis of a tumor. This is consistent with the reported range of 60-80% in the literature [1].

Endoscopic endonasal transsphenoidal surgery has gained wide acceptance over the last decade as an effective method to treat pituitary tumors; however, focused reports on using this modality to treat apoplectic patients are not abundant. Zhang, et al. [18] reported 41 patients (in a cohort of 59 cases) treated with an endoscopic transsphenoidal approach for hemorrhagic pituitary adenomas, although they did not describe the details of their surgeries. They did not break down the results based on surgical approach (microscopic versus endoscopic), but overall they achieved 90.8% GTR with >88% improvements of visual deficits. All of their patients were operated on within 24 hours of presentation. Chuang, et al. [9] reported their experience with 13 apoplectic patients (12 were treated with an endoscopic approach, 1 open craniotomy). They divided their patients into “uncomplicated” and “complicated” groups (with underlying medical comorbidities preventing earlier surgeries), the former had surgery within 3.5 days and the second group had surgery within 8.7 days. The first group had 100% vision improvement versus 50% in the second group. More patients in the second group needed long-term hormonal replacement compared to the first. They achieved GTR in eight out of the 12 patients treated with endoscopy. Hasegawa, et al. [17] reported three cases of endoscopic treatment of pituitary apoplexy in patients over 80. Endoscopic surgeries were chosen because they felt that this approach imparted less stress on the patients compared to an open craniotomy. Surgeries were performed 14-18 days after symptom onset and they achieved GTR in one patient (the other two they purposely elected for partial resection due to the concern for profuse bleeding from the cavernous sinus). All patients had a resolution of their visual symptoms and none needed postoperative hormonal replacement.

In our series, we achieved GTR in 90% of the patients, comparable to our success rate for non-apoplectic pituitary adenomas (90% for microadenomas and 67% for macroadenomas) [16]. We had one CSF leak (5%), which was managed conservatively. This patient was operated on in 2005 (earlier period of our series) at the beginning of our learning curve. Our CSF leak rate is now 0.7% for pituitary tumors [23]. For patients with visual disturbances, only 33.3% of patients had improvement in their visual field deficits. Improvement of subjective visual acuity decrease occurred in 66.7%, and 100% had a resolution of diplopia, consistent with the published trend of better reversal of ophthalmoplegic symptoms after surgical treatment of apoplectic patients [1-2]. In accordance with prior publications supporting early surgical intervention for better vision-related symptom reversal/improvement [6, 8-10], our data shows a lower rates of field cut improvement and overall later time-to-surgery (three of the four patients without improvement had surgery after one month of symptom onset). However, the total number of patients with documented visual field deficit in our series is too small to show a definitive trend of field cut improvement with early time-to-surgery. We have reviewed the published literature on microscope-based transsphenoidal surgery for pituitary apoplexy for comparison (Table 5). Most of the microscopic series focused on the effect of surgery on visual function. The extent of resection was infrequently reported, except one prior report documented a 63% rate of GTR [24], which was much lower than our 90% rate (Table 5). Likewise a 6-11% permanent DI rate is higher than that in our series (no new permanent DI) (Table 5). On the other hand, rates of improvement in visual field cuts were lower in our series (33.3% in ours versus >75% in the reported studies), and our time-to-surgery was significantly longer (Table 5). This was likely due to the late referral of patients to our institution for surgery rather than incomplete resection, given the high rates of GTR that we report. The relatively small number of patients with visual field defects in our population could also have skewed the result. Overall, these results argue for earlier referral for surgery in patients with a visual field deficit as well as the safety and efficacy of the endonasal approach for maximizing the extent of resection. Based on the relative infancy of the endoscopic technique, our follow-up time is shorter than that of the microscopic series. Continued collection of long-term follow-up data is warranted.

**Table 5:** Comparison of Surgical Results between Endoscopic and Microscopic Approach. C: complicated group, Desmo: desmopressin, E: endoscopic, M: microscopic, mo: months, NA: not available, OP: ocular palsy/diplopia, repl: replacement, Te: testosterone, TC: trans-cranial, Th: thyroid hormone, TS: transsphenoidal, St: steroid, Uncom: uncomplicated group, VA: visual acuity, VF: visual field, yr: year, “+”: improvement, “-“: worse , ^a^: plus other transnasal extended endoscopic approaches as needed.

Author & Year	Approach	Number of Patients Who Had Surgery	GTR	Length of Follow-up Mean (Range)	Visual Outcome	Endocrine Outcome (need of repl)	Complications
Bills, 1993	M (TS)	36	NA	2.8 yr	VA: +88% VF: +95% OP: +100%	Te/Th/St: 64%/89%/82% Desmo: 11% (another 9% transient need)	NA
Randeva, 1999	M (TS)	31	NA	6.3 yr (0.5 - 11)	VA: +86% VF: +76% OP:+ 91%	Te/Th/St: 43%/45%/58% Desmo: 6% (another 10% transient need)	NA
Sibal, 2004	M: 24 (TS), 3 (TC)	27	NA	49 mo (1 - 267)	VA: +93% VF: +93% OP: +93%	19% were normal postop (same at preop), 1 recovered from panhypopituitarism	NA
Lubina, 2005	M (TS)	34	63%	4.5 yr (2 mo - 17 yr)	VF: +81% OP: +71%	Panhypopituitarism: 27%, single axis: 40-79% Desmo: 8% (another 27% transient need)	2 mortality (within 6 mo)
Semple, 2005	M:48: (TS) 10: (TC)	58	NA	55.8 mo (1 mo - 22 yr)	VA: +85% VF: +94% -2% OP: +100%	Desmo: 9% (5/58) (another 3 (5%) transient need)	NA
Chung, 2006	M:1 (TC) E: 12 (TS)	13	66.6% (8/12)	22.5 mo (2 - 43)	Uncom: +100% C: +14.3 (1/7)	No “+” in hypocortisolism or DI patients, 1 “-“ with hypothyroidism, 3 “+” w/ hypogonadism	CSF leak: 8.3% (1/12)
Zhang, 2007	M (TS: 24) E (TS: 41)	65	90.8%	49 mo (median)	VA: +88.4% VF: +92.7%	83.3% “+” for functional adenomas Desmo: 6.2 % (4/65) (another 7 (10.7%) transient need)	CSF leak: 1.5 % (1/65) Mortality: 3.1% (2/65)
Hasegawa, 2011	E (TS)	3	33.3% (1/3)	6 mo - 3 yr	+100%	Desmo: 33.3% (1/3) transient need	NA
Current study	E (TS^a^)	20	90% (18/20)	22 mo (6 days - 72 mo)	VA: +66.7% VF: +33.3% OP: + 100%	Panhypopituitarism: +33.3% (3/9) Hypocortisolism: +60% (3/5) Prolactinemia: +88.9% (8/9) Desmo: 10% (2/20) transient need	CSF leak: 5% (1/20) Epistaxis 5% (1/20) meningitis/con-fusion: 10% (2/20)

There are limitations with our study. It is retrospective in nature and we do not have long-term follow-up information on some of the patients. A portion of our patients are followed by local referring physicians; hence, it was difficult to ensure a standard follow-up protocol for the evaluation of endocrine and visual functions of all the patients.  

## Conclusions

The endoscopic endonasal transsphenoidal approach is an effective modality to treat patients presenting with pituitary apoplexy, and the rate of GTR is high with minimal risk and a low rate of permanent DI. Delayed surgery may result in lower rates of visual field defect improvement, and thus, patients with field defects should be referred as soon as possible for surgery.
